# Multifaceted Roles of Tantalum in Promoting the Thermoelectric Performance of Mg_3_(Sb,Bi)_2_ Based Materials

**DOI:** 10.1002/advs.75768

**Published:** 2026-05-25

**Authors:** Guangmeng You, Jingdan Lei, Kai Xu, Yuntian Fu, Xueke Gu, Chengxiao Peng, Zhen Wang, Jing Chen, Kunpeng Zhao, Chao Wang

**Affiliations:** ^1^ Institute For Computational Materials Science School of Physics and Electronics Henan University Kaifeng China; ^2^ School of Physics and Electric Engineering Anyang Normal University Anyang China; ^3^ State Key Laboratory of Advanced Fiber Materials College of Materials Science and Engineering Donghua University Shanghai China; ^4^ School of Electronic Information Zhengzhou University of Light Industry Zhengzhou China; ^5^ State Key Laboratory of Metal Matrix Composites School of Materials Science and Engineering Shanghai Jiao Tong University Shanghai China

**Keywords:** carrier mobility, grain boundary scattering, Mg_3_(Sb,Bi)_2_, thermal conductivity, thermoelectric

## Abstract

Mg_3_(Sb,Bi)_2_ has emerged as one of the most promising thermoelectric materials with the potential to complement commercial bismuth telluride. However, its performance is still hindered by pronounced carrier grain‐boundary scattering, relatively high lattice thermal conductivity, and poor chemical stability. In this work, we reveal the multifaceted role of Ta in enhancing the thermoelectric performance of Mg_3_(Sb,Bi)_2_‐based materials. The incorporation of Ta not only increases carrier concentration through the donor effect, but also boosts carrier mobility by promoting grain growth and reducing the potential barrier. Meanwhile, Ta point defects together with metallic Ta precipitates induce strong phonon scattering, leading to a substantial reduction in lattice thermal conductivity. These synergistic effects yield outstanding TE performance, with a remarkably high *zT* plateau of 1.8 between 573 and 723 K, surpassing the performance of most previously reported Mg_3_(Sb,Bi)_2_ systems. Moreover, a TE module constructed by both p‐type and n‐type Zintl phases achieves a high conversion efficiency of 9.6% at *ΔT* of 460 K. This work takes an important step toward the practical application of Mg_3_Sb_2_‐based TE materials and devices.

## Graphical abstract

A multifunctional tantalum incorporation strategy enables simultaneous tuning of carrier concentration, mitigation of grain‐boundary scattering, and enhanced phonon scattering in Mg_3_(Sb,Bi)_2_. These synergistic effects markedly improve carrier mobility and suppress lattice thermal conductivity, yielding both high material *zT* and high module conversion efficiency.

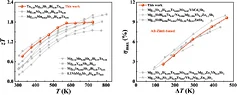

## 1 Introduction

Thermoelectric (TE) technology enables direct conversion between thermal and electrical energy via the Seebeck and Peltier effects. This solid‐state energy conversion offers a promising pathway for sustainable energy solutions, especially in applications involving waste heat recovery, remote sensors, wearable electronics, and solid‐state refrigeration [1, 2]. The performance of a TE material is characterized by its dimensionless figure of merit, *zT* = *S* ^2^ *σT*/*κ*, where *S* is the Seebeck coefficient, *σ* the electrical conductivity, *T* the absolute temperature, and *κ* the thermal conductivity. A higher *zT* indicates better TE efficiency; however, achieving high *zT* values remains challenging due to the intrinsically coupled nature of the transport parameters [3, 4, 5]. Conventional TE materials like Bi_2_Te_3_ [6], PbTe [7], and GeTe [8] offer high performance but suffer from scarcity (Te), toxicity (Pb), and thermal instability. These issues hinder large‐scale application, prompting the search for Te‐free alternatives using abundant, environmentally benign elements to achieve both sustainability and high TE efficiency.

Mg_3_(Sb,Bi)_2_, as a representative Zintl thermoelectrics, has recently emerged as a leading Te‐free TE material for near‐room‐temperature applications, offering a promising alternative to traditional Bi_2_Te_3_‐based systems [9, 10]. It crystallizes in a layered CaAl_2_Si_2_‐type structure and contains two distinct magnesium sites, where Mg1 is octahedrally coordinated and Mg2 is tetrahedrally coordinated [11]. This atomic configuration underpins its unique bonding environment and contributes to favorable transport properties. The electronic structure is characterized by a high valley degeneracy (*N_v_* = 6) at the conduction band minimum and a strongly anisotropic valence band maximum. A key advantage of this system is the tunable conduction type. Under Mg‐deficient conditions, intrinsic Mg vacancies (*V*_Mg_) serve as acceptor defects and stabilize p‐type conduction. Conversely, introducing excess Mg can effectively promotes the formation of Mg interstitials, thereby enabling n‐type conduction. By introducing aliovalent dopants on the cation sites (such as Sc [12], Y [13], La [14], Ce [15], Tm [16], Gd [17]) or on the anion sites (such as S [18], Se [19], Te [20]), the electron concentration of n‐type Mg_3_(Sb,Bi)_2_ can be driven up to ∼10^20^ cm^−3^. This markedly boosts the electrical transport, enabling a peak *zT* exceeding 1.5 at around 750 K.

Despite these merits, several critical challenges remain for Mg_3_(Sb,Bi)_2_. Near room temperature, the electron transport in Mg_3_(Sb,Bi)_2_ is considerably hindered by the grain boundary (GB) scattering, particularly in fine‐grained polycrystalline samples [21]. Using 3D atom probe tomography, Kuo et al. revealed that the high potential barriers at grain boundaries was due to local Mg depletion. Strategies such as high‐temperature sintering [22], liquid‐phase sintering [23], and melting recrystallization [24] have been explored to enlarge the grain size. However, these approaches often suffer from high energy consumption, poor controllability, and complex grain‐boundary textures, making reproducibility and large‐scale fabrication challenging. Additionally, despite its intrinsic lattice anharmonicity, the lattice thermal conductivity of Mg_3_(Sb,Bi)_2_ remains well above the theoretical minimum. Common phonon‐suppression methods like doping [25, 26], nanostructuring [21], and composite engineering [27] risk concurrent electron scattering. Moreover, the chemical instability of Mg, including its high vapor pressure, reactivity with moisture, and propensity to volatilize, complicates synthesis and device reliability [28]. Notably, most optimization methods tend to address one issue at a time, making the holistic design of stable, high‐performance Mg_3_(Sb,Bi)_2_‐based materials an ongoing challenge.

In this work, we achieve a synergistic optimization of electrical and thermal transports, along with an enhancement of thermal stability, by incorporating tantalum (Ta) into Mg_3_(Sb,Bi)_2_. Among various transition metals, Ta is particularly attractive due to its high valence state, low solubility, and excellent chemical stability, which makes it a promising candidate for simultaneously tuning carrier concentration, grain boundary properties, and phonon scattering. First, a trace of Ta enters the crystal lattice and act as donor dopants, increasing the electron concentration. Second, Ta effectively promotes grain growth, thereby reducing GB scattering. Third, Ta modifies the GBs to lower the GB potential barrier and enhances carrier mobility. Fourth, the presence of nanoscale Ta‐rich secondary phases introduces effective phonon scattering centers, leading to a reduction in the lattice thermal conductivity. Finally, Ta addition improves the material's resistance to degradation during thermal cycling by stabilizing the microstructure and suppressing magnesium volatility. These combined effects offer a promising strategy for designing high‐performance, stable Mg_3_(Sb,Bi)_2_‐based TE materials and devices.

## 2 Results and Discussion

**FIGURE 1:**
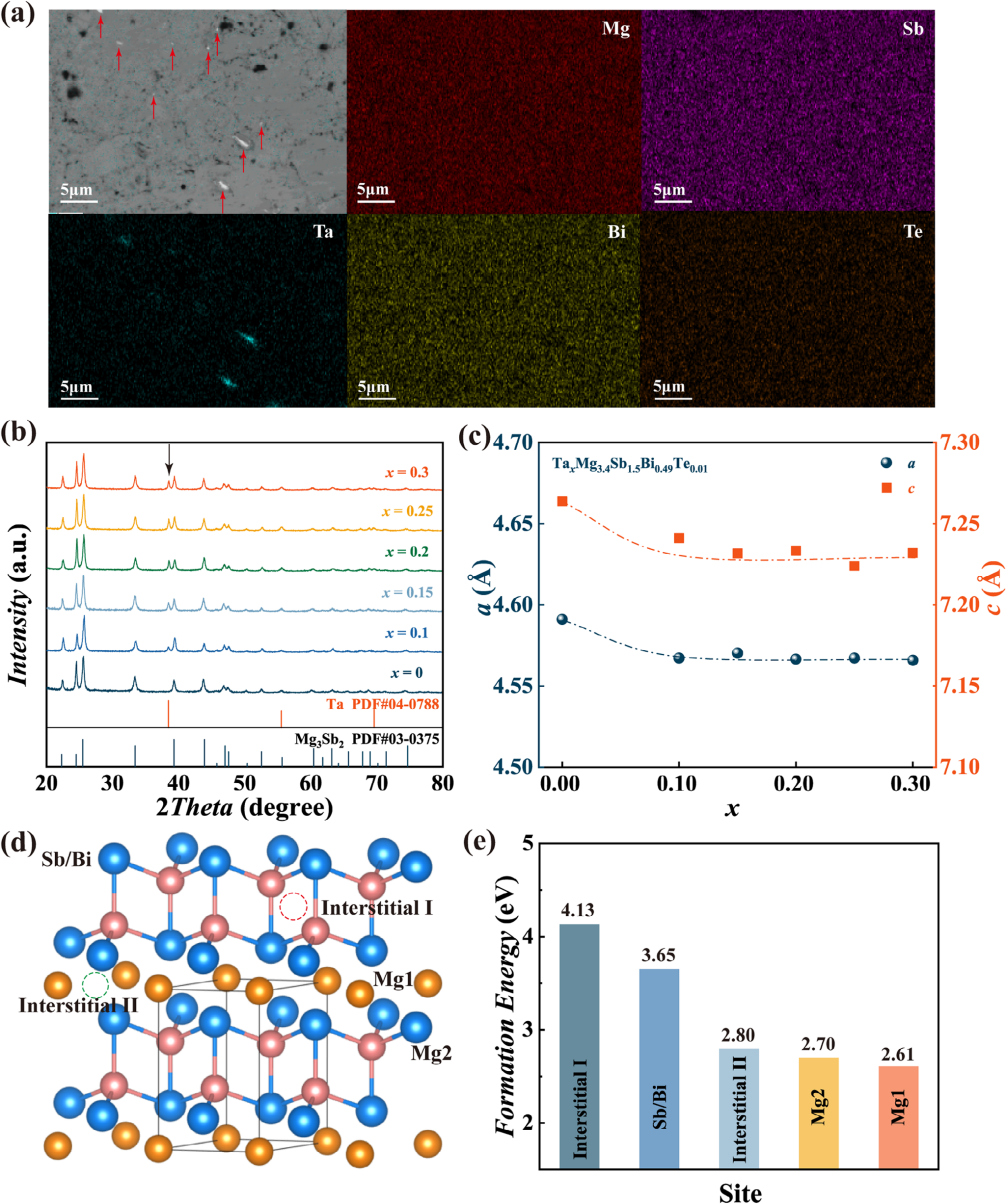
Crystal structure and microstructure characterization. (a) Back scattering electron (BSE) images and corresponding energy dispersive spectroscopy (EDS) mappings for *x* = 0.25 sample. (b) Room temperature X‐ray diffraction (XRD) patterns of Ta*_x_* Mg_3.4_Sb_1.5_Bi_0.49_Te_0.01_ (*x* = 0, 0.1, 0.15, 0.2, 0.25, 0.3) samples. (c) Evolution of lattice parameters (a‐axis and c‐axis) as a function of Ta doping concentration (*x*). (d) Schematic representation of the Mg_3_(Sb,Bi)_2_ crystal structure with intrinsic defects, illustrating the possible Ta occupancy at Mg vacancy sites. (e) Defect formation energies of Ta dopants at different sites in the Mg_3_(Sb,Bi)_2_ system, calculated using first‐principles calculations.

We fabricated a series of Ta*_x_* Mg_3.4_Sb_1.5_Bi_0.49_Te_0.01_ (*x* = 0, 0.1, 0.15, 0.2, 0.25, 0.28, 0.3) samples via ball milling technique. In this design, Bi serves to tailor the band gap and suppress lattice thermal conductivity, trace Te is doped to optimize the carrier concentration, while excess Mg is added to compensate for inevitable Mg loss during synthesis. Energy‐dispersive X‐ray spectroscopy (EDS) (Figure 1a) reveals that Mg, Sb, Bi, and Te are homogeneously distributed throughout the matrix, whereas Ta is predominantly segregated at grain boundaries as nano‐ to micro‐scale ribbons or dots. This finding demonstrates that the majority of Ta is present as discrete metallic particles in Mg_3_(Sb,Bi)_2_. The X‐ray diffraction (XRD) patterns of all samples are presented in Figure 1b. All the primary diffraction peaks can be well indexed to the trigonal structure (space group: *P* $\bar{3}$ m1) of Mg_3_(Sb,Bi)_2_. Notably, even a small addition of Ta induces a distinct metallic Ta diffraction peak at 2θ ≈ 38.5°, which is consistent with the EDS observations. Rietveld refinement results (Figure 1c) show a slight contraction in lattice parameters when *x* < 0.1, followed by negligible changes beyond this doping level, suggesting that the solubility limit of Ta is likely below 0.1. The lattice shrinkage is attributed to the partial substitution of larger Mg^2+^ ions (72 pm) by smaller Ta^5+^ ions (64 pm). The X‐ray photoelectron spectroscopy (XPS) results also confirm the presence of both Ta^5+^ and metallic Ta (Figure S1). Ta may occupy five possible lattice sites: two nonequivalent Mg sites, one Sb/Bi site, and two distinct interstitial sites (Figure 1d). First‐principles calculations of defect formation energies (Figure 1e) reveal positive values for all Ta doping sites, with Ta substitution at the Mg1 site exhibiting the lowest formation energy. This finding indicates that the dissolved Ta preferentially occupies the Mg1 site from a thermodynamic standpoint.

**FIGURE 2:**
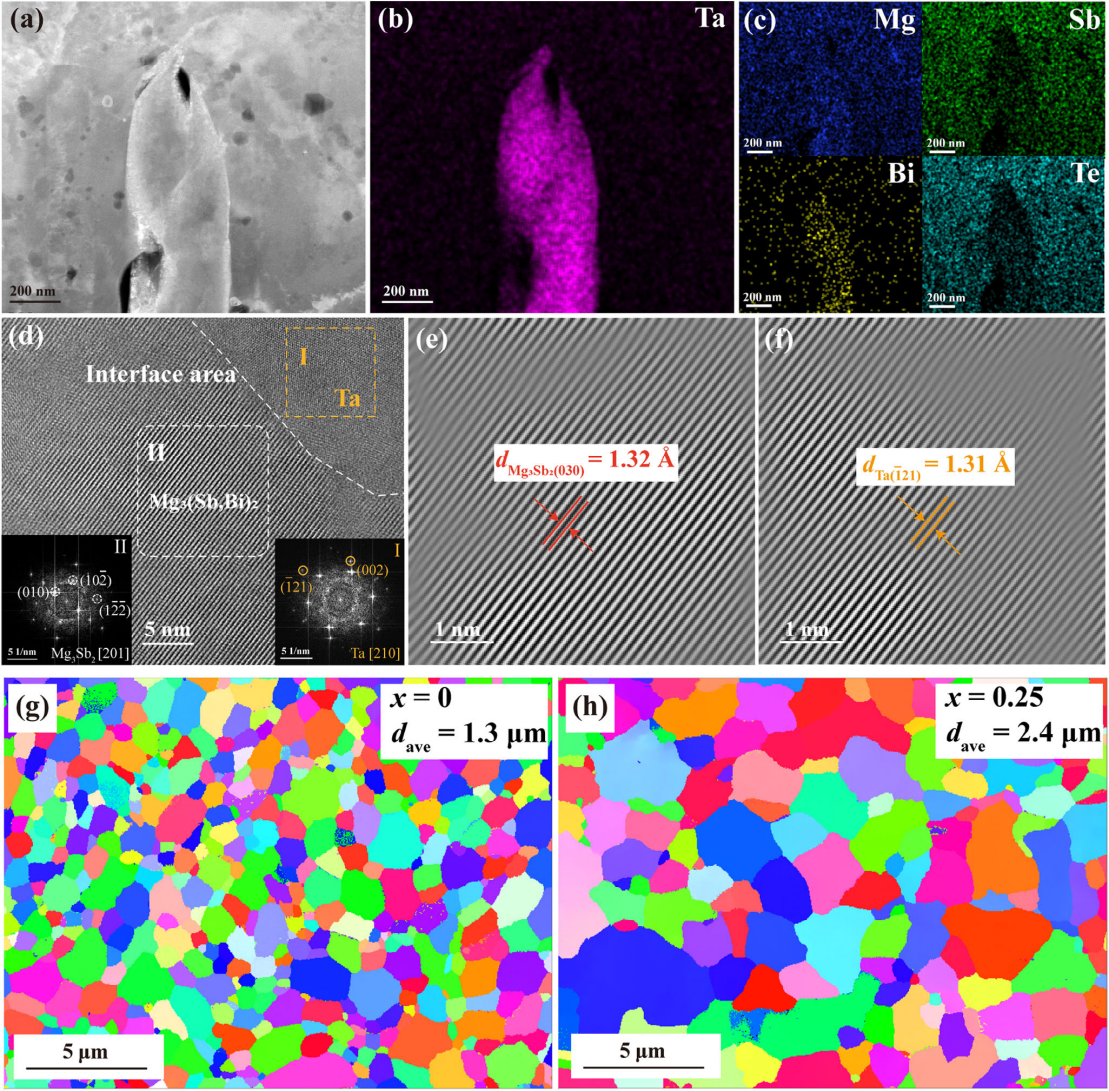
Microstructural and compositional characterization of Ta‐incorporated Mg_3_(Sb,Bi)_2_. (a) TEM image of a Ta‐rich region. (b) Corresponding energy dispersive spectroscopy (EDS) mappings of Ta, and (c) Mg, Sb, Bi, and Te. (d) High‐resolution TEM image of the interface between Mg_3_(Sb,Bi)_2_ and Ta, with the selected area electron diffraction (SAED) patterns indexed to Mg_3_Sb_2_ [201] and Ta [210], respectively. (e,f) Lattice fringes of Mg_3_Sb_2_ (030) with a spacing of 1.32 Å and Ta (‐121) with a spacing of 1.31 Å. (g,h) Electron backscatter diffraction (EBSD) images of samples with *x* = 0 and *x* = 0.25.

To further investigate the microscopic state of undissolved Ta and its influence on grain boundary (GB) structures, we conducted transmission electron microscopy (TEM) analysis. As shown in Figure 2a, significant enrichment of Ta was observed at the grain boundaries. This Ta‐rich region is accompanied by co‐segregation of Mg and Bi, in contrast to the Mg‐deficient GBs reported by Kuo et al. [29]. The local enrichment of Ta and Mg is expected to lower the GB potential barrier for electron transport. Similar compositional features at GBs have been previously reported [30, 31]. High‐resolution TEM imaging (Figure 2b) reveals a coherent interface between the metallic Ta and Mg_3_(Sb,Bi)_2_ matrix. The corresponding fast Fourier transform (FFT) patterns can be well indexed to the [210] plane of metallic Ta and the [201] plane of Mg_3_(Sb,Bi)_2_, respectively. Inverse fast Fourier transform (IFFT) further reveals that the (030) interplanar spacing of the Mg_3_(Sb,Bi)_2_ matrix (1.32 Å, Figure 2e) closely matches the (‐121) lattice spacing of Ta (1.31 Å, Figure 2f). The well‐aligned interface suggests strong interfacial bonding and good lattice matching, which are favorable for electron transport across grain boundaries.

The grain size evolution was analyzed using electron backscatter diffraction (EBSD) technique. As shown in Figure 2g,h and Figure S2, the average grain size increases from ∼1.3 µm in the Ta‐free sample to ∼2.4 µm in Ta_0.25_Mg_3.4_Sb_1.5_Bi_0.49_Te_0.01_ sample, indicating that Ta addition promotes grain growth. Similar phenomenon has been also observed in Nb and Mo doped Mg_3_(Sb,Bi)_2_, which can be attributed to two possible mechanisms [30, 32]. First, the metallic Ta phase may act as a heterogeneous nucleation site during sintering, facilitating grain growth of Mg_3_(Sb,Bi)_2_ phase via a seeding effect. Such phenomenon is commonly observed in melting, sintering, crystallization, or recrystallization processes. Second, the Ta inclusions may generate localized stress fields that interact with grain boundaries, potentially inducing curvature or destabilization of otherwise flat boundaries. Such effects can lower the energy barrier for GB migration, thereby accelerating the coalescence of small grains and promoting the formation of larger grains [33].

**FIGURE 3:**
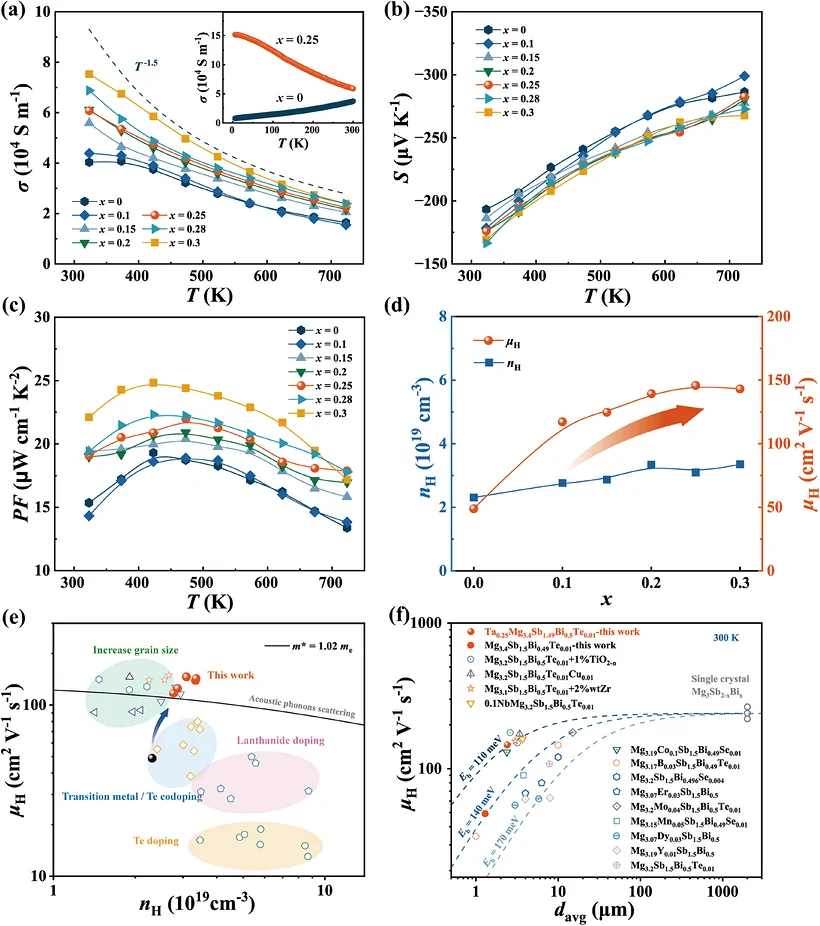
Electrical transport properties. Temperature dependent (a) Electrical conductivity *σ*, (b) Seebeck coefficient *S*, and (c) Power factor *PF* of the Ta*_x_* Mg_3.4_Sb_1.5_Bi_0.49_Te_0.01_ samples. The inset of Figure (a) shows the electrical conductivity of the samples at *x* = 0 and *x* = 0.25 over the temperature range of 5–300 K. (d) The relationship between Hall carrier concentration and mobility for the samples in this work and literatures in the Mg_3_Sb_1.5_Bi_0.5_‐based system [10, 34, 35, 36, 37, 38, 39]. (e) The carrier concentration dependent Hall mobility for Mg_3_Sb_1.5_Bi_0.5_ with a comparison to reported data. (f) The relationship between *µ_H_* and grain size under constant effective mass and similar carrier concentration [22, 32, 36, 37, 38, 41, 42, 43, 44, 45, 46, 47, 48, 49, 50].

The presence of partially dissolved Ta and undissolved Ta metallic phases plays a critical role in modulating the electrical properties of Ta*_x_* Mg_3.4_Sb_1.5_Bi_0.49_Te_0.01_. As shown in Figure 3a, the electrical conductivity *σ* of all samples exhibits a negative temperature dependency at high temperatures, characteristic of typical semiconducting behavior. However, notable differences emerge in the temperature dependency at low temperatures across samples with varying Ta content. Near room temperature, the *σ* of the Ta‐free sample (*x* = 0) shows a much weaker temperature dependency. Notably, below 300 K, it even exhibits a positive temperature dependency (see the inset in Figure 3a), in stark contrast to the negative dependency observed for the Ta‐containing samples. The distinct *σ* behavior of the Ta‐free sample is attributed to the smaller grain size and enhanced GB scattering, which result in thermally activated transport behavior below room temperature. At 5 K, the electrical conductivity of the Ta‐free sample is only 0.79 × 10^4^ S m^−1^. With increasing Ta content, *σ* increases significantly. For example, the *x* = 0.25 sample reaches a conductivity of 15.16 × 10^4^ S m^−1^ at 5 K, which is an order of magnitude higher than that of the Ta‐free sample. Moreover, the temperature dependency of *σ* in Ta‐containing samples progressively approaches the ideal *T* ^−1.5^ trend, indicative of reduced GB scattering. With increasing Ta content, the Seebeck coefficient *S* gradually decreases (see Figure 3b), which is attributed to the increase of carrier concentration. The negative sign of *S* confirms that electrons are the dominant charge carriers. The Pisarenko analysis reveals that the experimental *S* values for all samples align well with a theoretical curve with an effective mass of 1.02 *m_e_* (Figure S3), indicating that Ta incorporation does not significantly alter the electronic band structure near the Fermi level. The calculated power factors (*PFs*) are shown in Figure 3c. Across the entire temperature range, Ta‐containing samples exhibit higher *PFs* compared to the Ta‐free sample, with peak values occurring around 450 K. The *x* = 0.3 sample achieves a maximum *PF* of 24.8 µW cm^−1^ K^−2^, which is about 30% higher than that of the Ta‐free counterpart (19.2 µW cm^−1^ K^−2^), and also exceeds the *PF* values reported for many Mg_3_Sb_1.5_Bi_0.5_‐based materials in the literature (Figure S4).

To gain further insight, Hall measurements were conducted at room temperature to determine the carrier concentration (*n_H_*) and carrier mobility (*µ_H_*). As shown in Figure 3d. The carrier concentration increases modestly with Ta content, rising from 2.3 × 10^19^ cm^−3^ for the *x* = 0 sample to 3.4 × 10^19^ cm^−3^ for the *x* = 0.3 sample, suggesting that the dissolved Ta acts as a donor. More notably, the carrier mobility *µ_H_* also exhibits a substantial enhancement, increasing from 49 cm^2^ V^−1^ s^−1^ (*x* = 0) to 145 cm^2^ V^−1^ s^−1^ (*x* = 0.25), which is the dominant factor driving the observed improvement in *σ*. A comparison with other reported Mg_3_Sb_1.5_Bi_0.5_‐based materials (Figure 3e) highlights the superior *µ_H_* of our Ta‐doped samples [10, 34, 35, 36, 37, 38, 39]. It is well established that, near room temperature, the carrier transport in Mg_3_(Bi, Sb)_2_ is significantly influenced by the GB scattering. According to the GB‐dominated transport model proposed by Seto [40], the carrier mobility governed by GB scattering can be described by

$$\mu_{\mathrm{GB}}=de{\left(\frac{1}{2\pi m^{*}k_{B}T}\right)}^{\frac{1}{2}}exp\left(-\frac{E_{b}}{k_{B}T}\right)$$

where *d* is the grain size, m^*^ is the carrier effective mass, *k_B_* is the Boltzmann constant, and *E*_b_ is the height of the potential barrier. This equation indicates that increasing *d* or reducing *E*_b_ is essential for enhancing mobility. Accordingly, the observed improvement in *µ_H_* for the Ta‐doped samples can be attributed to two synergistic effects: (1) grain growth promoted by Ta addition, which reduces GB density; and (2) Ta‐induced GB modification, which lowers *E*_b_ and facilitates electron transport. Figure 3f illustrates the relationship between *µ_H_* and grain size under constant effective mass and similar carrier concentration, based on the GB‐dominated transport model (see Supporting Information for details) [22, 32, 36, 37, 38, 41, 42, 43, 44, 45, 46, 47, 48, 49, 50]. As observed, the carrier mobility *µ_H_* gradually increases with grain size, particularly when the grain size is below 10 µm. However, the large enhancement of *µ_H_* observed in our Ta‐containing samples cannot be attributed solely to grain growth. The Ta‐free sample exhibits a barrier height *E*_b_ of ∼140 meV. In contrast, Ta‐doped samples exhibit a reduced *E*_b_ of ∼110 meV, comparable to that of samples with grain boundaries modified by Cu or Zr [42, 43]. Li et al. also reported that Nb induced metal–semiconductor interfacial can lower the GB barrier [45]. The barrier height *E*_b_ at GBs is known to be governed by interfacial trapping states, ionized impurities, and dielectric properties [51]. Here, the Ta secondary‐phase interfacial layer and coherent interfaces alter local chemistry and defect distributions, resulting in a lower grain boundary barrier height. These findings underscore that second‐phase‐assisted GB engineering is an effective strategy to lower potential barriers.

**FIGURE 4:**
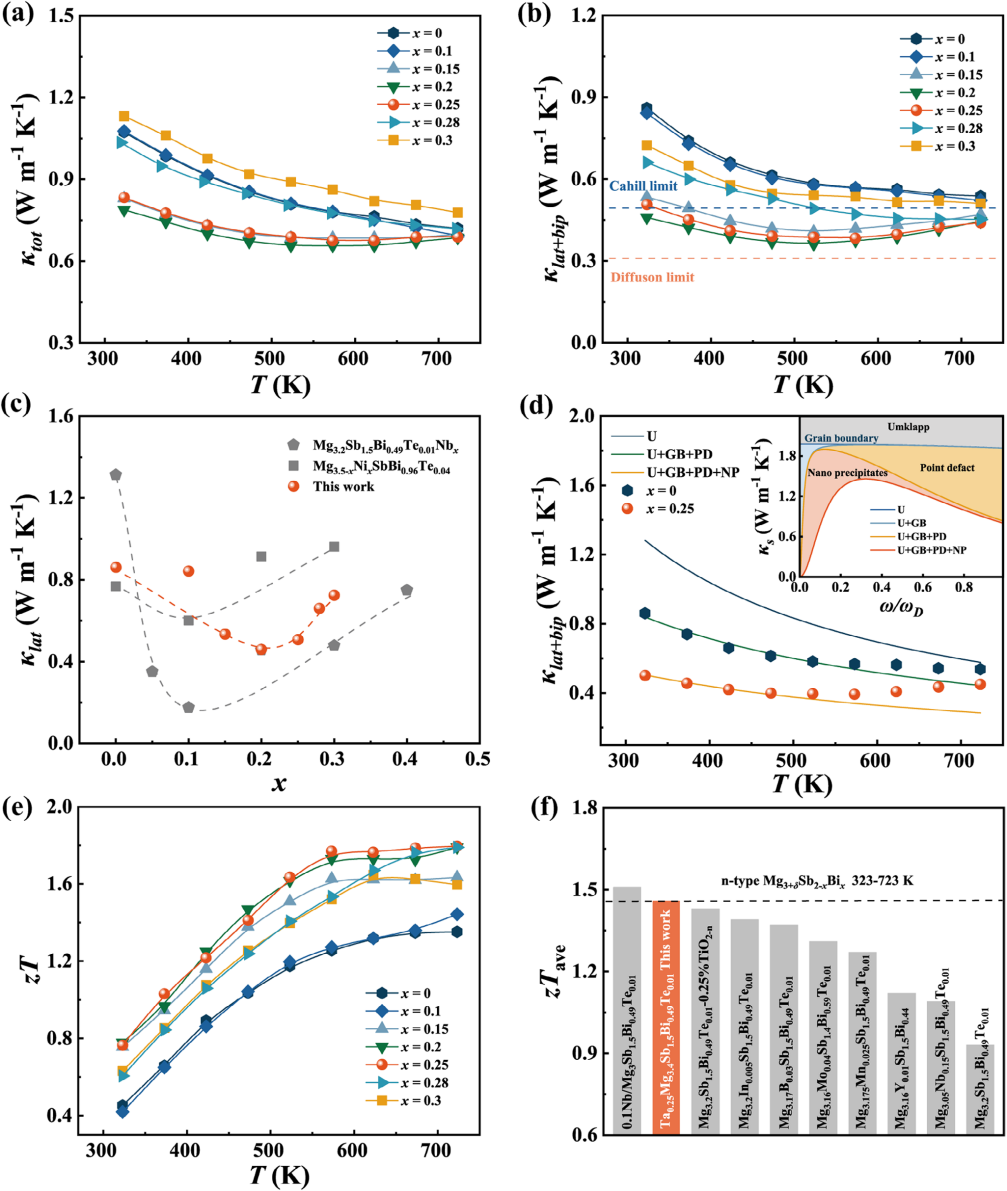
Thermal properties and figure of merit *zT*. Temperature dependent (a) Total thermal conductivity *κ*. (b) Lattice thermal conductivity and bipolar thermal conductivity (*κ_lat+bip_*) for Ta*_x_* Mg_3.4_Sb_1.5_Bi_0.49_Te_0.01_ (*x* = 0 – 0.3) samples. (c) Lattice thermal conductivity as a function of doping content *x* near room temperature [54, 55]. (d) Debye–Callaway model fitting for the *x* = 0 and *x* = 0.25 sample, where (U) represents phonon–phonon scattering, (GB) represents grain boundary scattering, (PD) represents point defect scattering, and (NP) represents nanoprecipitate scattering. The inset shows calculated spectral lattice thermal conductivity, with contributions from different scattering mechanisms. (e) Figure of merit *zT* for Ta*_x_* Mg_3.4_Sb_1.5_Bi_0.49_Te_0.01_ samples. (f) Average *zT* within 323–723 K of our samples with a comparison to previously reported Mg_3_Sb_1.5_Bi_0.5_‐based materials [10, 20, 32, 35, 37, 41, 45, 47, 56].

The incorporation of Ta also plays a significant role in tailoring the thermal transport properties of Ta*_x_* Mg_3.4_Sb_1.5_Bi_0.49_Te_0.01_. The total thermal conductivity (*κ_tot_*) of all samples is shown in Figure 4a. With increasing Ta content, *κ_tot_* initially decreases and then increases, reaching a minimum of 0.79 W m^−1^ K^−1^ at 323 K for *x* = 0.2 sample, which is substantially lower than that of the Ta‐free counterpart (1.07 W m^−1^ K^−1^). The total thermal conductivity comprises three components, i.e. lattice thermal conductivity (*κ_lat_*), bipolar thermal conductivity (*κ_bip_*), and electronic thermal conductivity (*κ_e_*) (Figure S5). For Ta*_x_* Mg_3.4_Sb_1.5_Bi_0.49_Te_0.01_, the bipolar contribution is negligible below 450 K. Therefore, by subtracting *κ_e_* from *κ_lat_*, the lattice contribution near room temperature can be reliably obtained (Figure 4b). Similar to *κ_tot_*, the *κ_lat_* also decrease initially with increasing Ta content before rising again beyond *x* > 0.2 (Figure 4c). The reduction in *κ_lat_* at lower doping levels is attributed to enhanced phonon scattering from point defects introduced by Ta substitution, as well as additional scattering from Ta metallic inclusions. For instance, at 323 K, *κ_lat_* decreases from 0.86 W m^−1^ K^−1^ in the Ta‐free sample to 0.46 W m^−1^ K^−1^ for *x* = 0.2, representing a 46% reduction. Notably, at 523 K, the minimum value of *κ_lat_* for the *x* = 0.2 sample drops to as low as 0.38 W m^−1^ K^−1^, which falls below the Cahill's limit and approaches the diffuson transport regime (see Supporting Information). The Cahill's limit corresponds to the minimum thermal conductivity under the assumption of strongly scattered phonons with short mean free paths, while the diffuson limit represents the extreme case of fully non‐propagating vibrational modes [52, 53]. Ththe e *κ_lat_* of Ta‐doped sample falls between the Cahill's limit and diffusion limit indicates that the material is in an intermediate transport regime, where both strongly scattered propagating modes and diffusive vibrational modes coexist. However, further increasing the Ta content beyond *x* = 0.2 leads to an anomalous rise in *κ_lat_*, likely due to the inherently high thermal conductivity of metallic Ta. When the contribution of the Ta's high thermal conductivity outweighs the reduction caused by phonon scattering, the *κ_lat_* will increases. Similar phenomenon has also been observed in Ni‐ and Nb‐doped Mg_3_(Sb,Bi)_2_ systems [54, 55] (Figure 4c).

To further elucidate the mechanisms by which Ta suppresses heat transport, we analyzed the *κ_lat_* using Debye–Callaway model (details provided in the Supporting Information). This model incorporates various phonon scattering mechanisms, including phonon–phonon Umklapp (U) scattering, grain boundary (GB) scattering, point defect (PD) scattering, and scattering from nanoscale precipitates (NP). As shown in Figure 4d and Figure S6, the experimental data are well captured only when both point defects and Ta nanoprecipitates are accounted for in the modelling. Frequency‐resolved thermal conductivity analysis reveals that Ta‐induced point defects primarily scatter high‐frequency phonons, while Ta nanoprecipitates are more effective at scattering low‐ to mid‐frequency phonons (see the inset in Figure 4d). This dual scattering effect contributes synergistically to the ultralow *κ_lat_* observed in the Ta‐added samples (Figure S7).

Benefiting from the enhanced *PF* and reduced *κ*, our Ta*_x_* Mg_3.4_Sb_1.5_Bi_0.49_Te_0.01_ samples exhibit excellent TE performance across the entire temperature range of 323–723 K. A similarly high level of *zT* values was observed for samples with *x* = 0.2 and 0.25 (Figure 4e). Notably, the near‐room‐temperature *zT* value increases from 0.45 for the Ta‐free sample to 0.78 for the *x* = 0.25 sample. At 573 K, the *zT* further rises to 1.8 and remains this value up to 723 K. As a result, a high average *zT* of 1.46 is achieved over the temperature range of 323–723 K, outperforming most previously reported Mg_3_(Sb,Bi)_2_‐based TE materials (Figure 4f) [10, 20, 32, 35, 37, 41, 45, 47, 56].

In addition to its excellent TE properties, Ta*_x_* Mg_3.4_Sb_1.5_Bi_0.49_Te_0.01_ samples also demonstrate remarkable reproducibility and thermal stability (Figures S8–S10). As shown in Figures S8 and S9, three independently synthesized batches of *x* = 0.25 and *x* = 0.3 samples exhibit highly consistent performance. Electrical cycling tests between 323 and 723 K (∼6 h per cycle) reveal that Ta‐containing samples retain ∼90% of their initial electrical conductivity after 13 cycles, while the Ta‐free counterpart suffers severe degradation, maintaining only ∼15% after 7 cycles (Figure S10). We speculate that Ta‐induced heterogeneous interfaces and structural integrity may suppress Mg migration along the grain boundaries, thereby enhancing the thermal stability of the material. More extensive and long‐term measurements are necessary to rigorously establish long‐term stability, which will be an important direction for future work.

**FIGURE 5:**
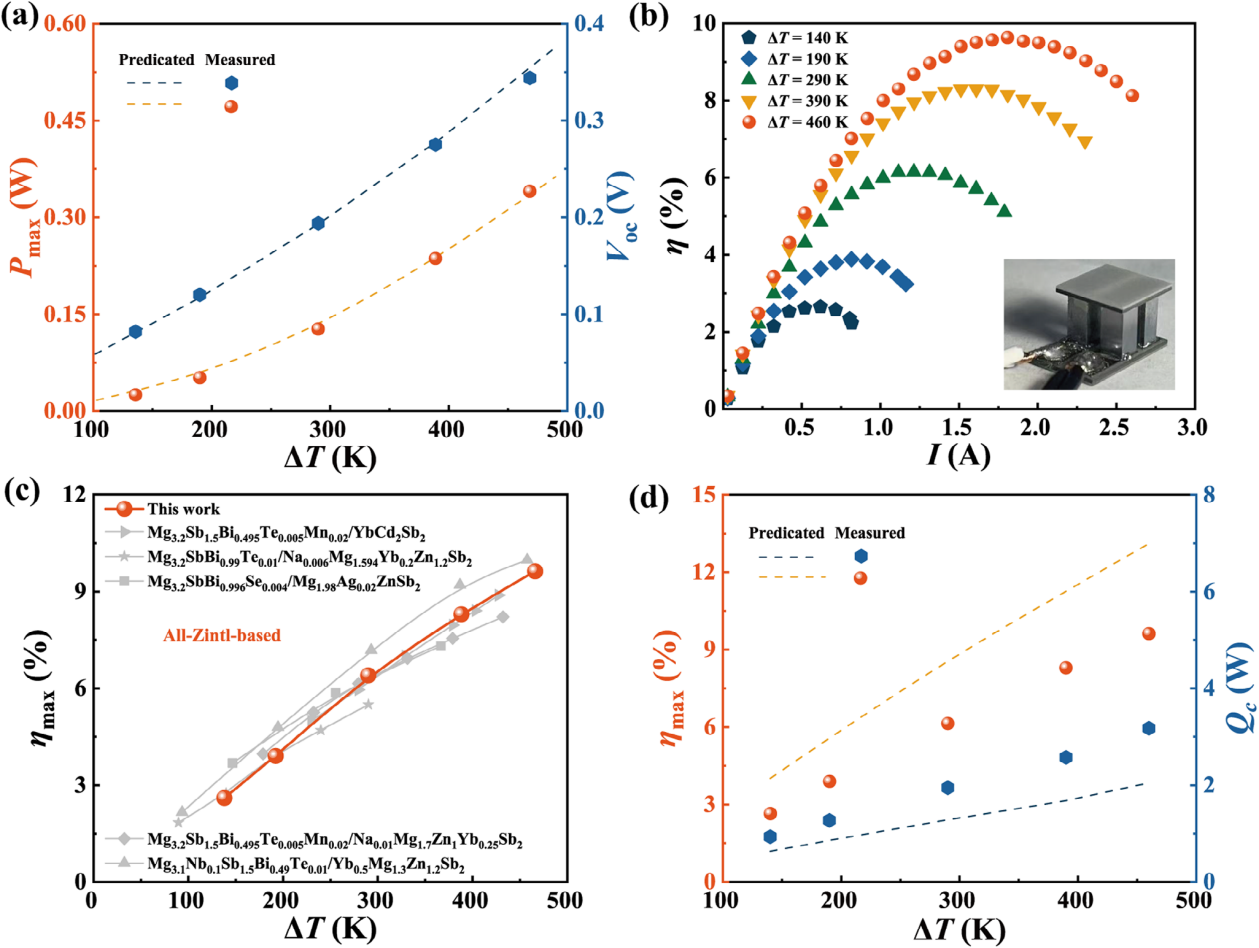
Performance of the TE module. (a) Output power (*P*_max_) and open‐circuit voltage (*V*_oc_) under different temperature gradients. The symbols represent the experimental measurements, while the dashed lines correspond to predicated values by finite‐element simulations. (b) Measured conversion efficiency *η* as a function of current under different temperature differences. (c) Comparison of the maximum conversion efficiency (*η_max_*) of our module as a function of *ΔT* with those of previously reported all‐Zintl‐based TE modules [9, 57, 58, 59, 60]. (d) Measured and predicted maximum efficiency (*η_max_*) and heat flow (*Q_c_*) under different temperature gradients.

We furtherly fabricated a 2‐pair TE module using Ta_0.25_Mg_3.4_Sb_1.5_Bi_0.49_Te_0.01_ as the n‐type leg and Mg_0.9_Zn_1.4_Yb_0.7_Sb_2_Li_0.003_ as the p‐type counterpart. Previous studies have identified Mg_2_Ni and Ni as low‐resistance and chemically stable interfacial layers, and they were therefore adopted as the interfacial materials for the n‐type and p‐type legs, respectively. A one‐step sintering process was employed to consolidate the interfacial layers and TE legs into a sandwich‐type architecture. A transient liquid‐phase bonding (TLPB) technique was subsequently used to join the Ni‐plated Cu electrodes to the legs, as detailed in the Experimental Section. The TLPB technique provides reliable, low‐temperature joining while ensuring robustness at elevated service temperatures. Linear output voltage‐current curves under various temperature gradient *ΔT* affirmed their good functions (Figure S11). Both the output power and open‐circuit voltage increase monotonically with *ΔT*, and the measured values closely follow the theoretical predictions (Figure 5a), indicating minimal contact resistance and strong interfacial bonding enabled by the optimized joining strategy. Figure 5b shows the energy conversion efficiency (*η*) as a function of current at different *ΔT* values, where *η* likewise improves with increasing *ΔT*. A maximum *η* of 9.6% is achieved at a *ΔT* of 460 K, comparable to the best‐performing all‐Zintl modules reported to date (Figure 5c). Finite element simulations suggest that the theoretical maximum *η* could reach as high as 13.6% at *ΔT* = 460 K (Figure 5d). The discrepancy between the measured *η* and predicted *η* mainly arises from the difficulty of accurately quantifying heat flow and suppressing radiative losses at high temperatures.

## 3 Conclusions

In summary, we demonstrate that introducing tantalum into Mg_3_(Sb,Bi)_2_ enables a synergistic optimization of the electrical and thermal transport properties. Trace amounts of Ta act as donors to increase the carrier concentration, while the undissolved Ta promotes grain growth and thereby enhancing the carrier mobility. Meanwhile, the second phase Ta effectively scatters phonons, lowering the room‐temperature lattice thermal conductivity to 0.46 W m^−1^ K^−1^. This synergistic optimization results in a high *zT* value of 1.8 at 723 K and an average *zT* of 1.46 in Ta_0.25_Mg_3.4_Sb_1.5_Bi_0.49_Te_0.01_ sample. Furthermore, a TE module consisting of both n‐type and p‐type Zintls demonstrates a high conversion efficiency of 9.6% at a temperature difference of 460 K. Overall, our work offers a new avenue for optimizing the TE performance of Mg_3_(Sb,Bi)_2_‐based materials and advances their progress toward practical application.

## 4 Experimental Section

### 4.1 Materials Synthesis and TE Module Fabrication

High‐purity elemental precursors, including Mg (magnesium powder, 99.5%, Aladdin), Sb (antimony shot, 99.99%, Aladdin), Bi (bismuth shot, 99.99%, Aladdin), Te (tellurium shot, 99.99%, Aladdin), and Ta (tantalum powder, 99.9%, Aladdin), were weighed according to the nominal composition of Ta*_x_* Mg_3.4_Sb_1.5_Bi_0.49_Te_0.01_ (*x* = 0, 0.1, 0.15, 0.2, 0.25, 0.28, 0.3), with a slight excess of Mg added to compensate for volatilization during high‐temperature processing. The weighed powders were transferred into a stainless‐steel jar and sealed in an argon‐filled glovebox (O_2_< 0.01 ppm). Mechanical alloying was performed by high‐energy ball milling (MSK‐SFM‐2, Hefei Kejing Materials Technology Co. Ltd.) at 1000 rpm for 6 h. The as‐milled powders were then loaded into a graphite die and consolidated by spark plasma sintering (SPS‐211LX, Fuji Electronic Industrial Co. Ltd.) at 1023 K for 10 min under a uniaxial pressure of 50 MPa. P‐type Mg_0.9_Zn_1.4_Yb_0.7_Sb_2_Li_0.003_ were also prepared by a high‐energy ball milling process, details can be found in our previous work [61].

For the n‐type and p‐type TE legs, Mg_2_Ni and Ni were employed as diffusion barrier layers. The Mg_2_Ni was fabricated by ball milling Mg and Ni powders in stoichiometric proportions using a high‐energy mill at 1200 rpm for 12 h under an argon atmosphere. Both n‐type TE joints of Mg_2_Ni/Ta_0.25_Mg_3.4_Sb_1.5_Bi_0.49_Te_0.01_/Mg_2_Ni and p‐type TE joints of Ni/ Mg_0.9_Zn_1.4_Yb_0.7_Sb_2_Li_0.003_/Ni were fabricated by the one‐step SPS technique for 10 min, with a sintering temperature at 1023 and 923 K, respectively. The n‐type and p‐type TE legs were diced into dimensions of 3.1 × 3.1 × 7.6 and 2.4 × 2.4 × 7.6 mm^3^, respectively. The two pairs of TE modules had an overall size of 10 × 10 × 8.6 mm^3^. Nickel‐coated and copper‐plated aluminum nitride ceramic sheets, featuring excellent thermal conductivity, were selected as electrically isolated substrates at the hot and cold sides. The transient liquid phase bonding (TLPB) technique was utilized to join the TE legs to nickel‐plated copper electrodes, with SAC0307 solder paste serving as the bonding interlayer. The TLPB process was conducted at 563 K for 30 min under a protective argon atmosphere to ensure interfacial integrity. Four copper wires were soldered to the cold‐side for current and voltage measurements during module performance characterization.

### 4.2 Material and TE Module Characterization

The phase structures of the synthesized samples were characterized by X‐ray diffraction (XRD, Cu Kα radiation) over a 2θ range of 20°–80°. Microstructural and compositional analyses were carried out using transmission electron microscopy (TEM, JEM‐F200, JEOL Ltd.), field‐emission scanning electron microscopy (FESEM, JSM‐7001F, JEOL Ltd.), and energy‐dispersive X‐ray spectroscopy (EDS). X‐ray photoelectron spectroscopy (XPS, Thermo Scientific K‐Alpha) was further employed to analyze the chemical states of the constituent elements. Electrical conductivity (*σ*) and Seebeck coefficient (*S*) were measured simultaneously using a ZEM‐3 system (ULVAC RIKO, Inc.) under a low‐pressure helium atmosphere in the temperature range of 323–723 K. Total thermal conductivity (*κ*) was calculated according to *κ* = D*C_p_ρ*, where thermal diffusivity (D) was obtained via laser flash analysis (DLF‐1/EM1200, TA Inc.) and density (*ρ*) was determined from the measured dimensions and mass of the sintered samples. The heat capacity (*C_p_*) was estimated using the Dulong–Petit limit, which is commonly adopted for Mg_3_(Sb,Bi)_2_ at moderate to high temperatures due to its relatively low Debye temperature. Carrier concentration and mobility were measured at room temperature using a Hall effect system (ET9005, East Changing Technologies) under a constant magnetic field. The measurement uncertainties were estimated as ±3% for *S*, ±5% for *σ*, ±7% for *κ*, and ±15% for *zT*.

The TE conversion efficiency of the two pair module was evaluated using a home‐built test system, enabling precise temperature regulation even under a fluctuant temperature for hot‐side [62]. The measurements were conducted in an argon‐filled chamber, where a uniaxial pressure of 0.5 KPa was applied to minimize electrical and thermal contact resistances. The hot‐side temperature of the module was controlled by a heater, while the cold‐side temperature was maintained at approximately 288 K using circulating water. A LabVIEW‐based program was employed to regulate the input power of the heater for accurate temperature control. An external DC load was used for generating voltage–current (*V–I*) curves. The power generation efficiency (*η*) was calculated using the formula *η* = *P* / (*P* + *Q*_c_), where *P* denotes the output power, and *Q*_c_ represents the heat flow at the cold side. The *η* was measured under temperature differences (*ΔT*) of 140, 190, 290, 390, and 460 K.

### 4.3 Theoretical Calculations

First‐principles calculations were conducted within density functional theory (DFT) using the Vienna Ab‐initio Simulation Package (VASP) [63]. The exchange–correlation energy was described by the GGA‐PBE functional with the PAW method, and a plane‐wave cutoff of 450 eV was employed [64]. Defect formation energies of doped Mg_3_Sb_2_ were evaluated in a 3 × 3 × 2 supercell with dopants considered at Mg1, Mg2, Sb, interstitial, and cage sites. Brillouin zone integration was performed using a 3 × 3 × 2 Monkhorst‐Pack grid, and atomic positions were relaxed until forces were below 0.01 eV/Å. Convergence thresholds of 10^−5^ eV for energy and 10^−2^ eV/Å for force were adopted. Post‐processing and visualization were carried out using VASPKIT and VESTA, respectively.

The defect formation energy was calculated using the following equation [65]:

$$\Delta E_{f}^{\mathrm{defect}}=E_{\mathrm{defect}\;}\;-E_{\mathrm{perfect}\;}+qE_{F}+\sum_{i}n_{i}\mu_{i}$$

where *E_perfect_* and *E_defect_* represent the total energies of the pristine and defective supercells, respectively. *q* denotes the charge state of the defect, with *q<0* indicating additional electrons and *q>0* indicating hole formation, while *E_F_* is the Fermi level. The term *n_i_* corresponds to the number of atoms of type *i* that are removed or added, and *µ_i_* represents the chemical potential of the respective atomic species.

## Conflicts of Interest

The authors declare no conflicts of interest.

## Supporting information




**Supporting File**:advs75768‐sup‐0001‐SuppMat.docx.

## Data Availability

The data that support the findings of this study are available from the corresponding author upon reasonable request.
