# Supplementary material for: Multifaceted Roles of Tantalum in Promoting the Thermoelectric Performance of Mg3(Sb,Bi)2 Based Materials
Source: Adv Sci (Weinh). 2026 May 25;13(46):e75768. doi: 10.1002/advs.75768 (PMC13335782; doi:10.1002/advs.75768)
Supplement: Supplementary file 1 [file ADVS-13-e75768-s001.docx]

Supplementary Materials for

**Multifaceted Roles of Tantalum in Promoting the Thermoelectric Performance of Mg_3_(Sb,Bi)_2_ Based Materials**

*Guangmeng You,^a^ Jingdan Lei,^a,b,*^ Kai Xu,^c^ Yuntian Fu,^c^ Xueke Gu,^a^ Chengxiao Peng,^a^ Zhen Wang,^a^ Jing Chen,^d^ Kunpeng Zhao,^e,*^Chao Wang,^a,*^*

**Calculation of the minimum lattice thermal conductivity**

The minimum lattice thermal conductivity $\kappa_{min}$ estimated by the simplified Cahill’s model for a normal solid can be expressed as ^1^:

$$\kappa_{min}=\frac{1}{2}{(\frac{\pi}{6})}^{1/3}kn^{2/3}(2v_{t}+v_{l})$$

where *v*_t_ is the transverse speed of sound, *v_l_* is the longitudinal speed of sound, and *n* is the number density of atoms. The data of sound speed are obtained from the reported work ^2^. Additionally, an assessment of the high temperature diffuson limit of the lattice thermal conductivity is made possible by the obtained sound velocities based on the following equation ^3^:

$$\kappa_{\mathrm{diff}}=0.76kn^{2/3}\frac{1}{3}(2v_{t}+v_{l})$$

**Thermal transport modeling**

The lattice thermal conductivity of alloys was calculated by the modified Debye-Callaway model, which can be expressed as: ^4, 5^

$$\kappa_{lat}=\frac{k_{B}}{2\pi^{2}v}{(\frac{k_{B}}{\hbar})}^{3}T^{3}\int_{0}^{\theta_{D}/T} \frac{x^{4}e^{x}}{\tau_{tot}^{-1}{(e^{x}-1)}^{2}}\text{ }dx$$

Where *ħ* is the reduced Planck constant, $\theta_{D}$ is the Debye temperature, *x* is the relation of *ħω*/*k*_B_T, *ω* is the phonon frequency, and $\tau_{tot}$ is the overall phonon scattering relaxation time, $v$ is the average sound speed. $\tau_{tot}$ can be attributed to five phonon scattering mechanisms in this work, including phonon-phonon Umklapp scattering (U), normal scattering (N), grain boundary scattering (GB), point defect scattering (PD), nano precipitates scattering (NP). The phonon scattering relaxation time for respective mechanism can be expressed as follows:

$${\text{τ}_{\text{tot}}}^{\text{-1}}\text{=}\text{τ}_{\text{U}}^{\text{-1}}\text{+}\text{τ}_{\text{N}}^{\text{-1}}\text{+}\text{τ}_{\text{GB}}^{\text{-1}}\text{+}\text{τ}_{\text{PD}}^{\text{-1}}\text{+}\text{τ}_{\text{NP}}^{\text{-1}}$$

$\tau_{U}$ are given by

$$\tau_{U}^{-1}=\frac{\hbar\gamma^{2}}{2\pi\bar{M}v_{s}^{2}\theta_{D}}\omega^{2}T\exp\left( -\frac{\theta_{D}}{3T} \right)$$

where *γ* is Grüneisen parameter, $\bar{V}$ and $\bar{M}$ are the average atomic volume and average atomic mass.

The frequency-independent $\tau_{GB}$ is defined by

$$\tau_{GB}^{-1}=\frac{v}{d}$$

where *d* is the mean grain size, obtained from the EBSD data.

The relaxation time $\tau_{PD}$ of point defect scattering can be expressed as

$$\tau_{PD}^{-1}=\frac{\overline{V}\omega^{4}}{4\pi^{3}v^{3}}*\sum(1-x_{i})[{(\frac{M_{i}-M}{M})}^{2}+\varepsilon{(\frac{a_{i}-a}{a})}^{2}]$$

The contribution of nano precipitates scattering to the relaxation time can be expressed as:

$$\tau_{NP}^{-1}=v\left( \sigma_{S}^{-1}+\sigma_{l}^{-1} \right)^{-1}V_{NP}$$

$$\sigma_{S}=2\pi R^{2}$$

$$\sigma_{l}=\frac{4}{9}\pi R^{2}\left( \frac{\Delta D}{D} \right)^{2}\left( \frac{\omega R}{v} \right)^{4}$$

In which, *R* is the precipitates average radius, *D* is the mass density of matrix materials, *ΔD* is the difference between the precipitates/pores and matrix materials. These fitted parameters are listed in **Table S1**.

The frequency dependence of the lattice thermal conductivity can be calculated as following model ^6^:

$$\kappa_{s}(\omega)=\frac{k_{B}}{2\pi^{2}v}\tau_{C}(\omega){(\frac{\hbar\omega}{k_{B}T})}^{2}\frac{\omega^{2}e^{\frac{\hbar\omega}{k_{B}T}}}{{(e^{\frac{\hbar\omega}{k_{B}T}}-1)}^{2}}$$

**Figure S1.** High-resolution XPS spectra of the Ta 4*f* core level.

**Figure S2.** Grain-size distribution of the *x* = 0 and *x* = 0.25 samples obtained from EBSD.

**Figure S3.** Experimental *S* vs. *n* values and calculated Pisarenko plot at 300 K. For comparison, data from Mo-doped ^7^, Co-doped ^8^, and Mg_2_Cu-doped ^9^ samples are included.

**Figure S4.** Comparison of the power factor with previous studies of Mg_3_Sb_1.5_Bi_0.5_-based materials^10-17^.

**Figure S5.** Temperature-dependent electronic thermal conductivity (*κ_e_*) of Ta*_x_*Mg_3.4_Sb_1.5_Bi_0.49_Te_0.01_ (*x* = 0, 0.1, 0.15, 0.2, 0.25, 0.3) samples.

**Figure S6.** Debye-Callaway model fitting results for the *x* = 0 and *x* = 0.25 samples, where (U) denotes phonon–phonon scattering, (GB) denotes grain-boundary scattering, (PD) denotes point-defect scattering, and (NP) represents nanoprecipitate scattering.

**Figure S7.** Temperature-dependent lattice thermal conductivity for *x* = 0.25 sample with a comparison to literature results for Mg_3_Sb_1.5_Bi_0.5_ ^10-17^.

**Figure S8.** Reproducibility tests of Ta_0.25_Mg_3.4_Sb_1.5_Bi_0.49_Te_0.01_ samples across the entire measurement temperature range.

**Figure S9.** Reproducibility tests of Ta_0.3_Mg_3.4_Sb_1.5_Bi_0.49_Te_0.01_ samples across the entire measurement temperature range.

**Figure S10.** Thermal cycling stability of the Ta_0.25_Mg_3.4_Sb_1.5_Bi_0.49_Te_0.01_ sample. Temperature-dependent **(a)** electrical conductivity *σ* and **(b)** Seebeck coefficient *S*. Stability of Ta*_x_*Mg_3.4_Sb_1.5_Bi_0.49_Te_0.01_ samples (*x* = 0 and 0.25) over the entire measurement temperature range: **(c)** normalized electrical conductivity *σ/σ_0_* and **(d)** Seebeck coefficient *S/S_0_*.

**Figure S11.** Measured output power (*P*) and voltage (*U*) as a function of current (*I*) at different temperatures.

**Table S1.** Parameters used for the Debye-Callaway model based on the various phonon scattering mechanisms.

| Parameters | Symbol | Values  (*x* = 0) | Values  (*x* = 0.25) |
| --- | --- | --- | --- |
| Debye temperature (K) | $\theta_{D}$ | 192 | 192 |
| Longitudinal sound velocity (m/s) | $v_{l}$ | 3730 | 3730 |
| Transverse sound velocity (m/s) | $v_{t}$ | 1820 | 1820 |
| Average sound velocity (m/s) | $v$ | 1970 | 1970 |
| Average grain size (m) | *R* | 1.3×10^-9^ | 2.4×10^-9^ |
| Grüneisen parameter | *γ* | 1.7 | 1.7 |
| Average atomic mass | $\bar{M}$*(Kg)* | 0.7×10^−25^ | 0.7×10^−25^ |
| Average atomic volume | $\bar{V}$*(m^3^)* | 1.9×10^-29^ | 1.9×10^-29^ |
| Number density of precipitates | *N_P_ (m^-3^)* |  | 2.15×10^22^ |
| Average radius for the precipitates | *R (m)* |  | 10×10^-9^ |
| Matrix density | *D (Kg m^-3^) (m)* |  | 4.10×10^3^ |
| Density difference between matrix and precipitates | $\Delta D$*(Kg m^-3^)(m)* |  | 6.0×10^3^ |

**References**

1.	D. G. Cahill, S. K. Watson and R. O. Pohl, *Phys. Rev. B*, 1992, **46**, 6131-6140.

2.	X. Shi, X. Zhang, A. Ganose, J. Park, C. Sun, Z. Chen, S. Lin, W. Li, A. Jain and Y. Pei, *Mater. Today Phys.*, 2021, **18**, 100362.

3.	M. T. Agne, R. Hanus and G. J. Snyder, *Energy Environ. Sci.*, 2018, **11**, 609-616.

4.	J. Callaway, *Phys. Rev.*, 1959, **113**, 1046-1051.

5.	J. Yang, G. Meisner and L. Chen, *Applied physics letters*, 2004, **85**, 1140-1142.

6.	T. Deng, T. Xing, M. K. Brod, Y. Sheng, P. Qiu, I. Veremchuk, Q. Song, T.-R. Wei, J. Yang, G. J. Snyder, Y. Grin, L. Chen and X. Shi, *Energy Environ. Sci.*, 2020, **13**, 3041-3053.

7.	L. Wang, N. Sato, Y. Peng, R. Chetty, N. Kawamoto, D. H. Nguyen and T. Mori, *Adv. Energy Mater.*, 2023, **13**, 2301667.

8.	B. Tian, H. Ma, X. An, Y. Mao, Q. Deng, Q. Sun and R. Ang, *Device*, 2024, **2**.

9.	J. Lei, K. Zhao, J. Liao, S. Yang, Z. Zhang, T.-R. Wei, P. Qiu, M. Zhu, L. Chen and X. Shi, *Nat. Commun.*, 2024, **15**, 6588.

10.	J.-W. Li, Z. Han, J. Yu, H.-L. Zhuang, H. Hu, B. Su, H. Li, Y. Jiang, L. Chen and W. Liu, *Nature Communications*, 2023, **14**, 7428.

11.	J. W. Li, H. Gao, Z. Han, J. Yu, H. L. Zhuang, L. Chen, H. Li, Y. Jiang, Z. Wang and Q. Zheng, *Advanced Materials*, 2025, 2503665.

12.	L. Wang, W. Zhang, S. Y. Back, N. Kawamoto, D. H. Nguyen and T. Mori, *Nat. Commun.*, 2024, **15**, 6800.

13.	X. Chen, J. Zhu, D. Qin, N. Qu, W. Xue, Y. Wang, Q. Zhang, W. Cai, F. Guo and J. Sui, *Science China Materials*, 2021, **64**, 1761-1769.

14.	L. Wang, N. Sato, Y. Peng, R. Chetty, N. Kawamoto, D. H. Nguyen and T. Mori, *Advanced Energy Materials*, 2023, **13**, 2301667.

15.	X. Chen, H. Wu, J. Cui, Y. Xiao, Y. Zhang, J. He, Y. Chen, J. Cao, W. Cai, S. J. Pennycook, Z. Liu, L.-D. Zhao and J. Sui, *Nano Energy*, 2018, **52**, 246-255

16.	Z. Liu, N. Sato, W. Gao, K. Yubuta, N. Kawamoto, M. Mitome, K. Kurashima, Y. Owada, K. Nagase and C.-H. Lee, *Joule*, 2021, **5**, 1196-1208.

17.	J. Li, F. Jia, S. Zhang, S. Zheng, B. Wang, L. Chen, G. Lu and L. Wu, *Journal of Materials Chemistry A*, 2019, **7**, 19316-19323.
